# Carbon-neutral energy cycles using alcohols

**DOI:** 10.1080/14686996.2018.1426340

**Published:** 2018-02-15

**Authors:** Takashi Fukushima, Sho Kitano, Shinichi Hata, Miho Yamauchi

**Affiliations:** ^a^ International Institute for Carbon-Neutral Energy Research, Kyushu University, Fukuoka, Japan

**Keywords:** Selective electrooxidation of alcohol, hydrogenation of carboxylic acid, fuel cell, electrolyzer, TiO_2_, 50 Energy Materials, 106 Metallic materials, 205 Catalyst / Photocatalyst / Photosynthesis, 206 Energy conversion / transport / storage / recovery, 207 Fuel cells / Batteries / Super capacitors

## Abstract

We demonstrated carbon-neutral (CN) energy circulation using glycolic acid (**GC**)/oxalic acid (**OX**) redox couple. Here, we report fundamental studies on both catalyst search for power generation process, i.e. **GC** oxidation, and elemental steps for fuel generation process, i.e. **OX** reduction, in CN cycle. The catalytic activity test on various transition metals revealed that Rh, Pd, Ir, and Pt have preferable features as a catalyst for electrochemical oxidation of **GC**. A carbon-supported Pt catalyst in alkaline conditions exhibited higher activity, durability, and product selectivity for electrooxidation of **GC** rather than those in acidic media. The kinetic study on **OX** reduction clearly indicated that **OX** reduction undergoes successive two-electron reductions to form **GC**. Furthermore, application of TiO_2_ catalysts with large specific area for electrochemical reduction of **OX** facilitates the selective formation of **GC**.

## Graphical abstract

**Figure:**
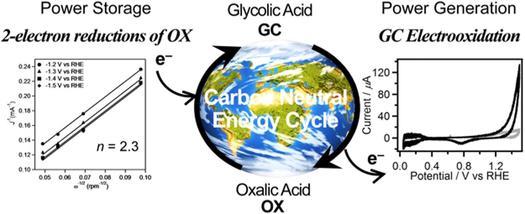

## 1. Introduction

Efficient power distribution is a key to realize a sustainable society driving with renewable energies. Hydrogen is believed to be the cleanest energy carrier due to no CO_2_ emission during combustion [1–5]. Industrial hydrogen, however, is mainly produced by reforming fossil fuels, such as, natural gas, propane, gasoline, diesel and so on. On the other hand, water electrolysis applied with electricity generated from renewable energies, such as photovoltaic, wind and hydraulic powers, i.e. renewable electricity, can provide carbon-neutral H_2_ [6–11]. However, fatal drawbacks of utilization of H_2_ as an energy carrier still remain. Namely, gaseous and chemically active H_2_ fold an infeasibility of widespread distribution due to its low volumetric energy density (13 MJ m^−3^), which makes difficulties in long-time storage and long-distance transport. H_2_ transport using a cryogenic liquid hydrogen tanker or a massive hydrogen pipeline system is expected to solve such problems but requests large cost. On the other hand, various H_2_ carriers such as organic hydrides [12], NH_3_ [13], amides [14] and formate converted from CO_2_ [15], have been proposed as a H_2_ carrier, and some have achieved the efficiencies demanded for practical use. In this regard, liquid energy carriers, e.g. gasoline, offer great merit by considering the manageability of liquid fuels. Thus far, we have proposed utilization of alcoholic compounds that are chemically stable and characterized with a high volumetric energy density, e.g. ethanol and ethylene glycol exhibit energy densities of are 23,000 and 22,000 MJ m^−3^, respectively. In the previous study, we succeeded in power generation from ethylene glycol by selective oxidation to oxalic acid (**OX**, (CO_2_H)_2_) with an alkaline type fuel cell employing Fe-group nanoalloy catalysts, i.e. CO_2_-free power generation on non-Pt catalysts [16–18]. The selective oxidation could produce 80% of potentially available electric power of ethylene glycol. Furthermore, we performed CO_2_-free power circulation using glycolic acid (**GC**, HOCH_2_-CO_2_H)/**OX** redox couple [19,20]. **GC** is a monovalent alcohol having 8600 MJ m^−3^ of an energy density in the case of selective oxidation as described in the following equation:

(1) $${\text{R-CH}}_{2}{\text{OH + H}}_{2}\text{O}\to \text{R}-{\text{COOH + 4H}}^{+}{\;\text{+ 4e}}^{-}.$$

Power generation was achieved via four-electron oxidation of **GC** into **OX** without CO_2_ emission on a Pt catalyst. More importantly, **GC** was reproduced via electrochemical hydrogenation of **OX** on an anatase TiO_2_ catalyst with hydrogen generated from water at a Pt anode. These results are the first demonstration of CO_2_-free power circulation using an alcohol/acid redox couple. Recently, direct solar energy storage was also achieved by applying photoanode for water oxidation [21].

Whereas we found out the excellent catalytic ability of TiO_2_ for **OX** reduction by testing the catalytic performance of various metals and their oxides in our previous paper [19], **GC** oxidation have been performed only with a Pt catalyst. Therefore, there is room for exploring novel catalysts for power generation from **GC**. Here, in this study, we examined the catalytic performances of various transition metals, including Ti, V, Fe, Co, Ni, Cu, Zn, Zr, Nb, Mo, Rh, Pd, Ag, Ta, W, Re, Ir, Pt, and Au, for the electrochemical oxidation of **GC**, and found that Pt exhibits the best catalytic performance and a few other noble metals have a potential to be an anode catalyst. Furthermore, we investigate catalytic properties of a carbon-supported Pt catalyst (Pt/C) and appropriate conditions for the electrochemical **GC** oxidation.

In our previous study, regeneration of **GC** was achieved by four-electron reduction of **OX**. However, we do not have any ideas about detailed mechanism, i.e. four-electron reduction is completed via one step reaction or successive two-electron reductions accompanied with formation of glyoxylic acid (HOOC–COH, GO)[19,22]. **OX** reduction has not been analyzed by electrochemical methods in detail. Knowledge of the reaction process will contribute to improvement of efficiency, reaction rate, product selectivity, and so on. We have found that crystallite phase of TiO_2_ is a decisive factor for **OX** reduction activity i.e. anatase-type TiO_2_ showed higher activities than that of rutile-type TiO_2_ [19], and expected that further investigation of the effects of other characteristics of TiO_2_ contributes to achieve high efficiency for the reaction system. In this study, we examined electrochemical analysis of **OX** reduction on TiO_2_ cathode and effects of specific surface area of TiO_2_ on performances of **OX** reduction using various anatase-type TiO_2_. We clarified that four-electron reduction of **OX** to **GC** proceeded through successive two-electron reductions and application of TiO_2_ catalysts with large specific area can suppress H_2_ production and led to high selectivity for reduction of **OX** to **GC**.

## 2. Experimental details

### 2.1. Materials

Glycolic acid (**GC**, 97.0%), oxalic acid (**OX**, 98.0%), potassium hydroxide (85.0%), and sodium sulfate (99.0%) were purchased from Wako (Osaka, Japan). Ethylene glycol was purchased from Kanto (Tokyo, Japan). 20 wt% Pt/C was purchased from Alfa Aesar (Ward Hill, MA, USA). Ti (99.5%), V (99.7%), Fe (99.99%), Co (99.9%), Ni (99%), Cu (99.9%), Zn (99.5%), Zr (99.2%), Nb (99.9%), Mo (99.95%), Pd (99.95%), Ag (99.98%), Ta (99.95%), W (99.95%), Au (99.95%) plates and Rh (99.9%), Re (99.97%), Ir (99.9%), Pt (99.98%) wires were purchased from Nilaco (Tokyo, Japan). Proton-conducting membrane (Nafion®, NRE-212) was purchased from Sigma-Aldrich Japan (Tokyo, Japan). Carbon felt (KURECA PAPER E-525) was purchased from KUREHA (Tokyo, Japan). All chemicals were used without further purification. A porous TiO_2_ sphere (PTS) was synthesized by a procedure reported previously [19]. A mixture of 1 mL of titanium tetrabutoxide, 10 mL of 2-propanol, and 30 mL of *N,N*-dimethylformamide were transferred into a 50 mL Teflon-lined autoclave and heated in an electric oven at 200 °C for 20 h. The product was collected by centrifugation and washed thoroughly with acetone and methanol. Obtained powder was calcined under air flow at 500 °C, and then PTS was synthesized. JRC-TIO-2, 7, 8, and 13 (Japan Reference Catalyst of TiO_2_, anatase-type TiO_2_ nanoparticles) were used as TiO_2_ samples.

### 2.2. Electrochemical studies

All electrochemical experiments were conducted using a three-electrode system connected to a VersaSTAT 4 potentiostat (Princeton Applied Research, Oak Ridge, TN, USA) or a HZ-7000 (Hokuto Denko, Tokyo, Japan). A coiled Pt wire (length 230 mm, diameter 0.5 mm, BAS Inc., Tokyo, Japan) was used as a counter electrode. An Ag/AgCl (RE-1B, BAS) or an Hg/HgO (RE-6A, BAS) reference electrode was used in acidic or alkaline conditions, respectively. Potentials applied to the working electrode were measured against a reference electrode and converted to the reversible hydrogen electrode (RHE) reference scale using:

$$E\;\left(\text{vs. RHE}\right)=E\;\left(\text{vs. Ag/AgCl}\right)+0.199\;\text{V}+0.059\;\text{V}\times \text{pH}$$

$$E\;\left(\text{vs. RHE}\right)=E\;\left(\text{vs. Hg/HgO}\right)+0.110\;\text{V}+0.059\;\text{V}\times \text{pH}$$

#### 2.2.1. Preparation of working electrodes

Ti, V, Fe, Co, Ni, Cu, Zn, Zr, Nb, Mo, Pd, Ag, Ta, W, Au plates (1 × 1 cm^2^) and Rh, Re, Ir, Pt wires (length 3 cm, diameter 0.5 mm) were used as a metal electrode for **GC** oxidation. Pt/C applied electrode was prepared using a commercial Pt/C (Alfa Aesar). An ethylene glycol suspension (0.225 g) of 20 wt% Pt/C was applied onto carbon felt (2 × 2 cm^2^). Japan Reference Catalyst of TiO_2_ (JRC-TIO) powders were used as electrode materials. A TiO_2_ electrode was prepared as follows. 4 mg of JRC-TIO-7 powder and 8 μL of 5 wt% Nafion solution were dispersed in 4 mL of 1:1 v/v water/isopropanol mixed solvent for longer than 30 min sonication to form a homogeneous ink. Then, 10 μL of the catalyst ink containing 10 μg of catalyst was loaded onto a rotating disk electrode (RDE) with 3 mm in diameter, i.e. 0.14 mg cm^−2^ of catalyst loading density. Ti plates (2 × 2 cm) were used as an electrode substrate after calcination at 450 °C for 30 min under air flow. JRC-TIO samples having a distinct specific surface area (JRC-TIO-2, 7, 8, 9, and 13) were chosen as TiO_2_ catalysts. A suspension of TiO_2_ powder (10 mg) in methanol (0.2 mL) was applied to the calcined Ti plate, and then, the TiO_2_-applied Ti plate was calcined at 500 °C for 1 h under flowing air.

#### 2.2.2. Cyclic voltammetry (CV) measurements

CV measurements were conducted by employing a three-electrode system connected to a VersaSTAT 4 potentiostat. An electrolyte aqueous solution (40 mL) was introduced into a glass cell (100 mL in volume). After the glass cell was tightly sealed with Teflon cap, Ar gas was bubbled for 30 min in order to purge the air from the inside of the cell. The current value was recorded against the applied potential with 10 mV/s scan rate and two scan cycles. The aqueous electrolyte solution containing 0.5 M Na_2_SO_4_ and 0.1 M (for metal electrodes) or 0.5 M (for Pt/C electrode) **GC** was used for acidic conditions, and the aqueous electrolyte solution containing 20 wt% KOH and 0.5 M **GC** was used for alkaline condition. The blank CV measurements were conducted with the electrolyte solution without **GC**. For acidic conditions, pH of the blank electrolyte solution was adjusted with H_2_SO_4_ to be identical to that of the corresponding electrolyte solution with **GC**.

#### 2.2.3. Linear sweep voltammetry measurements

Linear sweep voltammetry measurements were conducted by employing a three-electrode system connected to a HZ-7000 using RDE as a working electrode with varying rotating speed from 1000 rpm to 4000 rpm. An electrolyte aqueous solution (80 mL of 0.03 M **OX** and 0.2 M Na_2_SO_4_) was introduced into a glass cell (100 mL in volume, ALS Co., Ltd, Tokyo, Japan). After the glass cell was tightly sealed with Teflon cap, Ar gas was bubbled for 30 min to remove the air from the inside of the cell. The current value was recorded against the applied potential with 10 mV s^−1^ scan rate. The CV measurement for a blank solution was carried out by following the same procedures above-mentioned except for using electrolyte solution (80 mL of 0.2 M Na_2_SO_4_).

#### 2.2.4. Electrochemical oxidation of **GC** and product analysis

The electrochemical oxidation of **GC** at a constant potential, i.e. chronoamperometry (CA), was performed in a two-compartment electrochemical cell sealed to be gas-tight with Teflon caps. A piece of proton-conducting membrane was used as a separator. An aqueous electrolyte solution (0.5 M Na_2_SO_4_+0.5 M **GC** or 20 wt%, i.e. 3.56 KOH+0.5 M **GC** or 3.56 M LiOH+0.5 M **GC**, 38 mL) was introduced into an anodic cell (75 mL in volume) into which the working and reference electrode were subsequently immersed. A counter electrode was placed in a cathodic cell (75 mL in volume) containing an aqueous electrolyte solution (0.5 M Na_2_SO_4_ (pH adjusted to 2.3 with H_2_SO_4_) or 20 wt% KOH, 38 mL). After the Teflon caps were tightly closed, Ar gas was bubbled in both the anodic and cathodic cells for 30 min to purge the air from the cell. **GC** electrooxidation was conducted at 1.5 V vs. RHE and 50 °C for 2 h where the potential was controlled using a VersaSTAT 4 potentiostat. The reaction solution was collected from the anodic cell and analyzed using a high-performance liquid chromatograph (HPLC, LC-20AD, Shimadzu, Kyoto, Japan) equipped with a refractive index detector (RID-10A, Shimadzu). The gas was collected from the anodic cell and analyzed using gas chromatograph (GC, GC-8A, Shimadzu). In the case of alkaline condition, the electrolyte solution after CA experiment was neutralized with H_2_SO_4_, and generated CO_2_ was quantified by GC.

#### 2.2.5. Electrochemical reduction of **OX** and product analysis

CA experiments were conducted by employing a three-electrode system connected to a VersaSTAT4 potentiostat. The sample-modified Ti plate electrodes (2 × 2 cm) and RDE were used as a working electrode. The electrochemical reduction of **OX** at −0.7 V vs. RHE for Ti plate electrodes and −1.5 V vs. RHE for RDE was performed in a two-compartment electrochemical cell (75 mL volume for each). A piece of proton-conducting membrane was used as a separator. Electrolyte aqueous solutions (cathode: 40 mL of 0.03 M **OX** and 0.2 M Na_2_SO_4_, anode: 0.2 M Na_2_SO_4_) were introduced into the cells. The working/reference and counter electrodes were placed inside the cathode and anode cell, respectively. The pH value of the electrolyte solution in the cells was adjusted at 2.1 by adding H_2_SO_4_ solution. After the Teflon caps were tightly closed, Ar gas was bubbled both in cathode and anode cells for 30 min to purge the air from the cells. **OX** electrochemical reductions were conducted by controlling the working electrode potential at 50 °C. The reaction solution was collected from the cathode cell and analyzed using the HPLC.

#### 2.2.6. Definition of Faradaic yield

The Faradaic yield in the electrochemical experiments is defined by the following equation:

$$\text{Faradaic}\;\text{Yield}\;(\%)=\frac{m_{\text{products}}\times n\times F}{Q}\times 100,$$

where *m*_products_ is the moles of products; *n* represents the number of electrons required for the formation of products in **GC** oxidation (*n* = 2, 4, 1, and 3 for formation of glyoxylic acid, **OX**, HCOOH, and CO_2_, respectively) and **OX** reduction (*n* = 2 and 4 for formation of GO and **GC**, respectively); *F* is Faraday constant (96,485 C/mol); and *Q* is the total charge in Coulombs passed across the electrode during the electrolysis.

#### 2.2.7. Koutecky–Levich equation

Koutecky–Levich plots (*J* ^−1^ vs. *ω* ^−1/2^) were analyzed at various electrode potentials [23]. The slopes of linear fit lines were used to calculate the number of electrons transferred (*n*) on the basis of the Koutecky–Levich equation:

$$\frac{1}{J}=\frac{1}{J_{L}}+\frac{1}{J_{K}}=\frac{1}{B\omega^{1/2}}+\frac{1}{J_{K}}$$

$$B=0.62n\;F\;A\;C_{0}{(D}_{0}{)}^{2/3}v^{-1/6}$$

$$J_{K}=n\;F\;A\;k\;C_{0}$$

where *J* is the measured current density, *J*_*K*_ and *J*_*L*_ are the kinetic- and diffusion-limiting current densities, *ω* is the angular velocity, *n* is transferred electron number, *A* is the area of electrode/electrocatalyst, *C*_*0*_ is the bulk concentration of **OX**, *D*_*0*_ is the diffusion coefficient of **OX**, *ν* is the kinematic viscosity of the electrolyte.

## 3. Results and discussion

### 3.1. GC oxidation

#### 3.1.1. Catalytic activity test for transition metal electrodes

**Figure 1.**
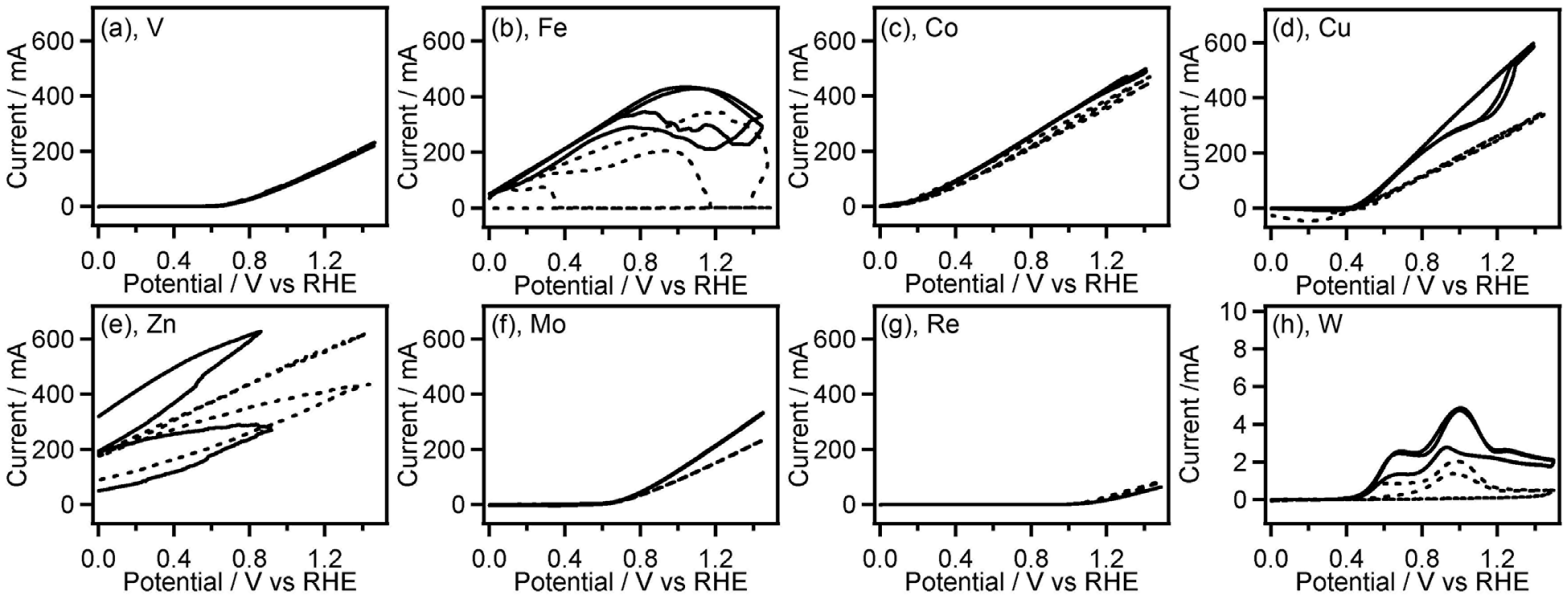
CVs (two-cycle scan) of V (a), Fe (b), Co (c), Cu (d), Zn (e), Mo (f), Re (g), and W (h) recorded in 0.1 M **GC**+0.5 M Na_2_SO_4_ (solid line) and 0.5 M Na_2_SO_4_ (broken line). The current range of graphs (a–g) is fixed to –50 to 700 mA.

**Figure 2.**
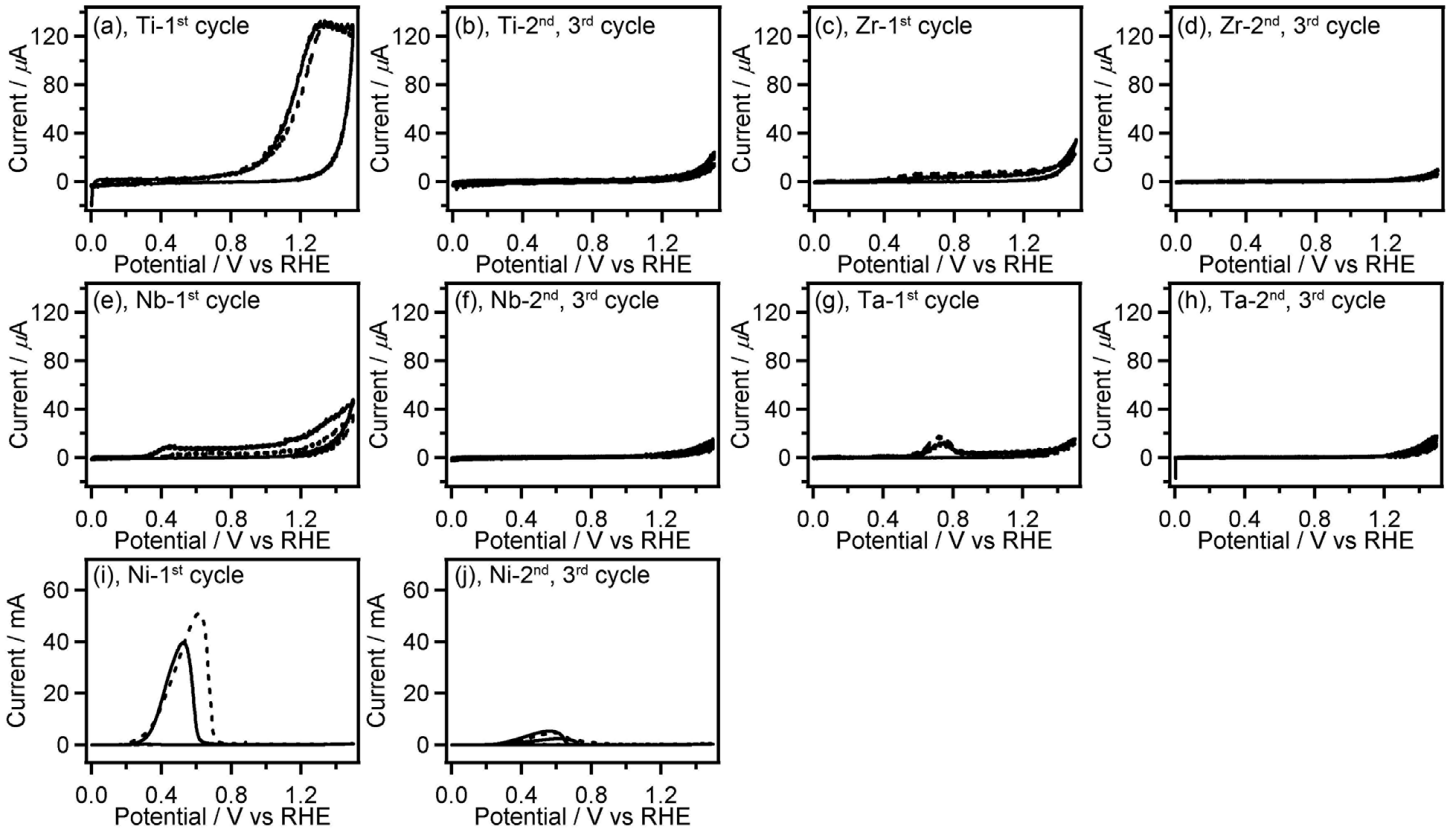
CVs (three-cycle scan) of Ti (a,b), Zr (c,d), Nb (e,f), Ta (g,h), and Ni (i,j) recorded in 0.1 M **GC**+0.5 M Na_2_SO_4_ (solid line) and 0.5 M Na_2_SO_4_ (broken line). The first (a,c,e,g,i) and the second, third (b,d,f,h,j) scan cycles of CVs are depicted separately. The current range of graphs (a–h) is fixed to –20 to 140 μA.

**Figure 3.**
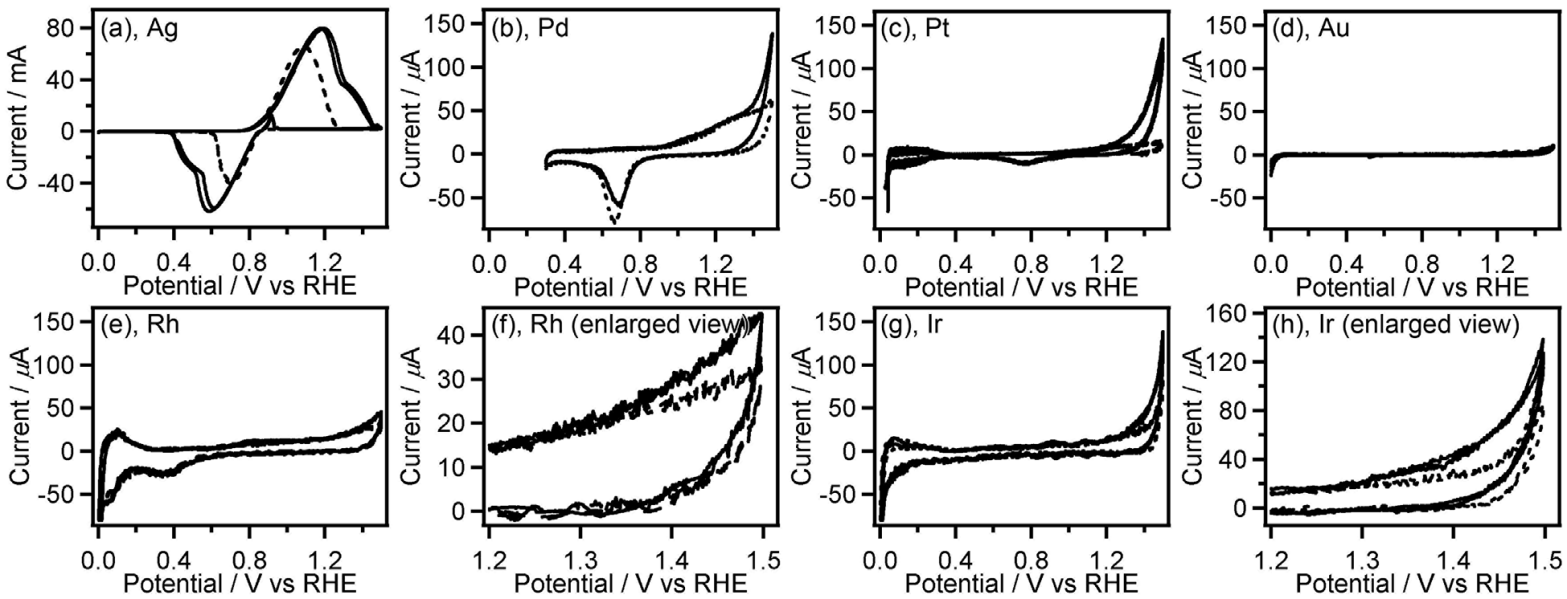
CVs (two-cycle scan) of Ag (a), Pd (b), Pt (c), Au (d), Rh (e,f), and Ir (g,h) recorded in 0.1 M **GC**+0.5 M Na_2_SO_4_ (solid line) and 0.5 M Na_2_SO_4_ (broken line). The current range of graphs (b–e,g) is fixed to –80 to 700 mA.

The catalytic test on a transition metal electrode for electrochemical oxidation of **GC** was conducted by performing cyclic voltammetry (CV) measurements using a metal plates of Ti, V, Fe, Co, Ni, Cu, Zn, Zr, Nb, Mo, Pd, Ag, Ta, W, and Au or wires of Rh, Re, Ir, and Pt as a working electrode in the presence and absence of **GC**. Figures 1–3 shows CVs recorded with the transition metal working electrodes in 0.5 M Na_2_SO_4_ aqueous solution containing and not containing 0.1 M **GC**.

**Figure 4.**
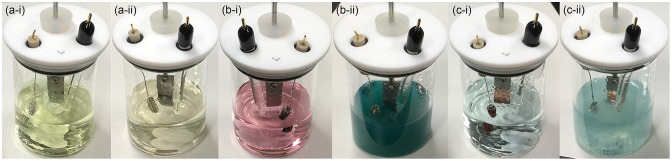
The colors of electrolyte solution after the CV measurements of Fe (a), Co (b), and Cu (c) electrodes in 0.1 M **GC**+0.5 M Na_2_SO_4_ (i) and 0.5 M Na_2_SO_4_ (ii).

Most of the non-noble metals such as V, Fe, Co, Cu, Zn, Mo, Re and W showed large anodic current (peak current: 5–700 mA) in their CVs (Figure 1). After the CV measurement of these metals, the surface of the metal plates lost luster and became dull, and the metal plates seemed to be smaller than their original size. Furthermore, the electrolyte solution after the CV measurements of Fe, Co, and Cu electrodes had characteristic colors (Figure 4) originated from their ions. These observations suggest that oxidation of these metals results in elution of the metal ion into the electrolyte solution. Interestingly, the anodic current obtained in CVs of Fe, Co, Cu, Zn, Mo, and W was increased in the presence of **GC**. Therefore, these metals may have certain ability to catalyze electrooxidation of **GC** although the elution of metal ions occurs, which is an unfavorable feature for the electrooxidation catalysts. On the other hand, other non-noble metals, i.e. Ti, Zr, Nb, Ta and Ni exhibited lower anodic current (peak current: 20 μA–60 mA) in the first cycle of the CVs, and the anodic current decreased drastically in the second CV cycle (Figure 2), suggesting the oxide film formation on the metal surface.

Among the noble metals such as Ag, Pd, Pt, Au, Rh and Ir, only Ag displayed the large anodic waves (peak current: 60–80 mA) in the forward (positive) scans of the CVs accompanied by the corresponding cathodic waves in the reverse (negative) scans (Figure 3(a)). It is widely known that electrooxidation of Ag in aqueous solution gives Ag_2_O film on the Ag surface, and electroreduction of resulting Ag_2_O affords the original Ag metal [24–26]. Therefore, the observed redox waves can be attributed to the Ag/Ag_2_O redox reaction. In this work, we used metal wires as electrode for CV measurement of Pt, Rh, Ir, and Re. The surface area of the metal wires is smaller than that of the metal plates (wires: ca. 0.47 cm^2^, plates: ca. 2 cm^2^). Even though we consider the smaller surface area of the metal wires, the anodic current in the absence of **GC** on the noble metals except for Ag, i.e. Pd, Pt, Au, Rh and Ir is very small (peak current: <80 μA, Figure 3(b)–(h)), which suggests the high oxidation resistance of these noble metals. In the CVs on the electrode of Rh, Pd, Ir, and Pt, the anodic current at potential more positive than 1.2 V vs. RHE was clearly increased in presence of **GC**, indicating electrochemical **GC** oxidation catalyzed on these metal electrodes. These results of CV measurements suggest that Rh, Pd, Ir, and Pt have preferable features, including high oxidation resistance and catalytic activity, for electrochemical oxidation of **GC**, and Pt shows the highest catalytic activity among all electrodes used here. We, hence, examined characteristics of Pt/C catalyzed electrochemical oxidation of **GC** in both acidic and alkaline media.

#### 3.1.2. Pt/C catalyzed electrochemical oxidation of GC

**Figure 5.**
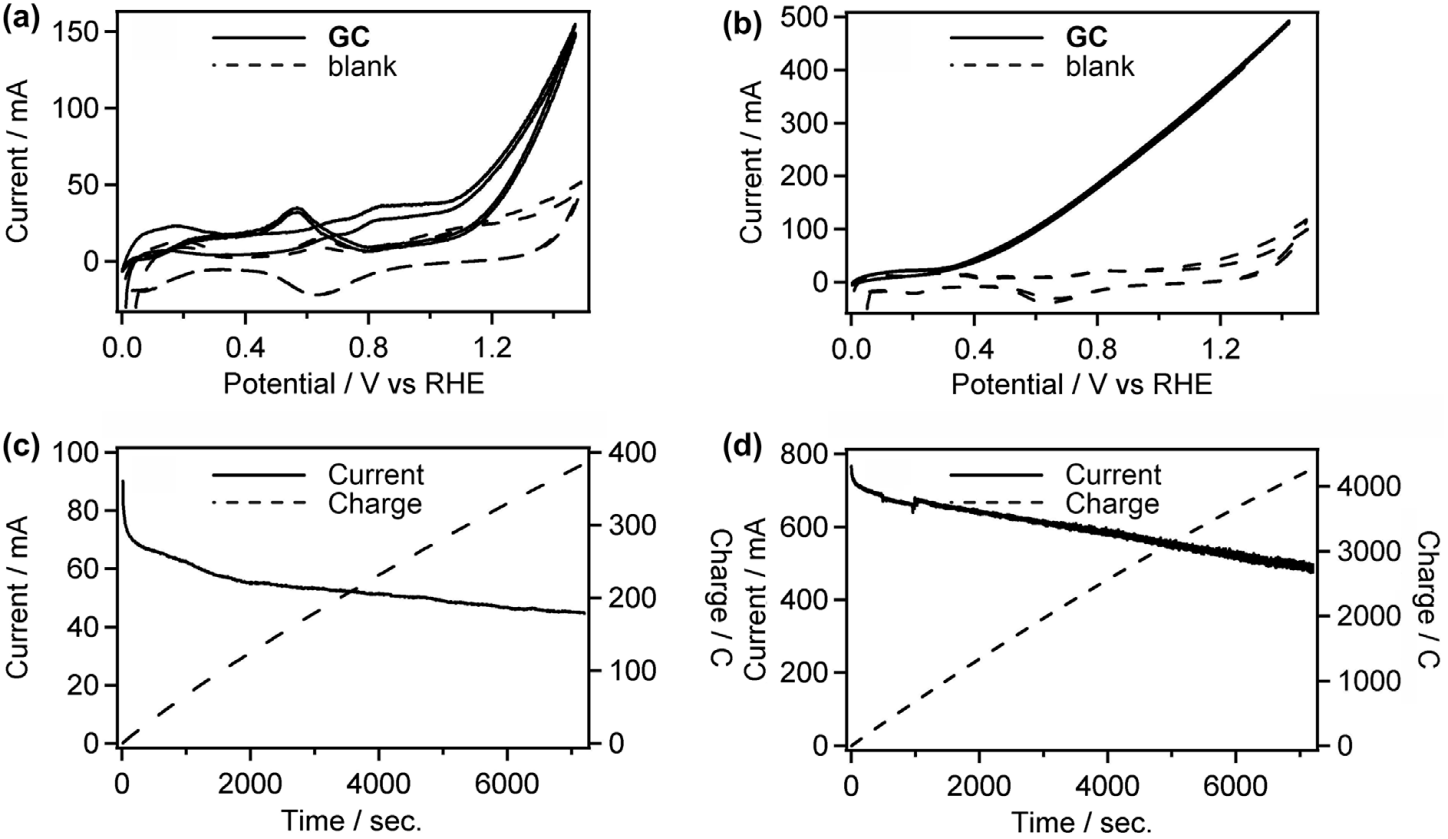
CVs of Pt/C in 0.5 M **GC**+0.5 M Na_2_SO_4_ (a) and 0.5 M **GC**+20 wt% KOH (b) at 50 °C. And CA curves of Pt/C in 0.5 M **GC**+0.5 M Na_2_SO_4_ (c) and 0.5 M **GC**+20 wt% KOH (d) at 50 °C.

Figure 5(a) and (b) compare CVs of Pt/C loaded carbon-felt electrode recorded in acidic (0.5 M **GC**+0.5 M Na_2_SO_4_, pH 2.3, (a)) and alkaline (0.5 M **GC**+20 wt% KOH, pH 14, (b)) media at 50 °C. The anodic current for the **GC** oxidation in acidic and alkaline media arose at 1.1 and 0.3 V vs. RHE, respectively, and increased constantly with potential to reach 150 and 500 mA at 1.5 V, respectively. Ample studies have demonstrated that the high concentration of OH^−^ in the electrolyte and adsorbed OH on the Pt surface greatly facilitate the de-protonation of alcohols, and thus significantly lower energy barrier of alcohol oxidation reaction [27–29]. This explains the substantial reduction of the onset potential and increase of catalytic current for the **GC** oxidation in alkaline media. In acidic solution, the catalytic current observed on the reverse (negative) scan was obviously lower than that obtained on the forward (positive) scan, and an anodic peak centered at 0.57 V was observed only in the reverse scan. (Figure 5(a)). This indicates that the catalyst was deactivated by the adsorption of CO generated by oxidative degradation of **GC** on the catalyst surface (*vide infra*), and the anodic peak around 0.6 V on the reverse scan is attributable to reactivation of the catalyst due to the desorption of CO.

**Figure 6.**
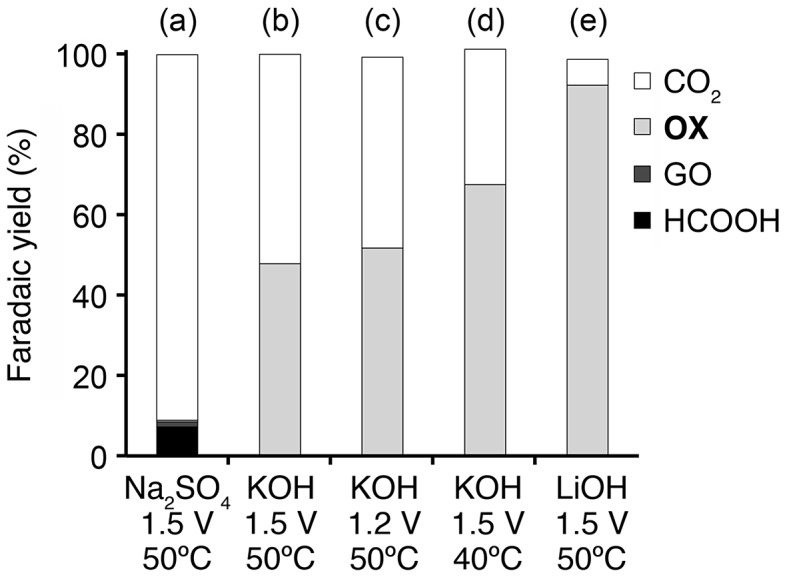
Faradaic yields for CO_2_, **OX**, GO, and HCOOH in **GC** electro-oxidation (a) at 1.5 V in 0.5 M **GC** and 0.5 M Na_2_SO_4_ at 50 °C, (b) at 1.5 V in 0.5 M **GC** and 20 wt%, i.e. 3.56 M KOH at 50 °C, (c) at 1.2 V in 0.5 M **GC** and 20 wt% KOH at 50 °C, (d) at 1.5 V in 0.5 M **GC** and 20 wt% KOH at 40 °C and (e) at 1.5 V in 0.5 M **GC** and 3.56 M LiOH at 50 °C.

**Scheme 1.**
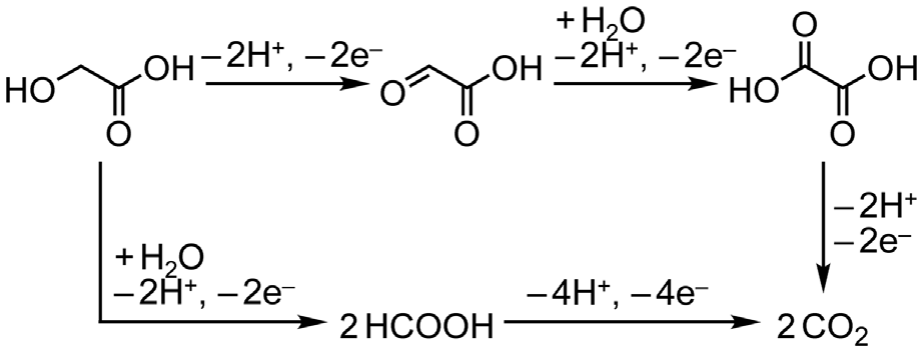
Proposed pathway for **GC** oxidation.

To elucidate the long-term stability of the Pt/C electrode and determine the oxidation products of **GC**, chronoamperometry (CA) experiments were performed in both acidic (0.5 M **GC**+0.5 M Na_2_SO_4_, pH 2.3) and alkaline (0.5 M **GC**+20 wt% KOH, pH 14) media at 50 °C. The applied potential of the CA experiments (1.5 V vs. RHE for both acidic and alkaline conditions) was decided from the CV results to obtain sufficient current necessary to products determination. Interestingly, Pt/C catalyst maintained the high activity (490‒770 mA) during the CA experiment in alkaline media (Figure 5(d)), while the catalyst was drastically deactivated in initial stage of the CA experiment, up to 80 s, and the electroreaction proceeded with the damaged catalyst (45‒65 mA) in acidic media (Figure 5(c)), Consequently, 385 and 4300 °C of charge passed across the electrode during the 7200 s of the CA experiment in acidic and alkaline media, respectively. Figure 6(a) and (b) show the products obtained in these CA experiments and Faradaic yield for each product, and Scheme 1 illustrates the pathway for electrooxidation of **GC** as inferred from the literature describing the electrochemical ethylene glycol oxidation [27–33]. The main products in **GC** oxidation in acidic solution were CO_2_ and formic acid (90.9 and 7.2% Faradaic yield, respectively). The preferential formation of the C_1_ compounds indicates the accelerated occurrence of the C‒C bond cleavage in the reacting molecules followed by further oxidation of the C_1_ intermediates generating adsorbed CO [27,32]. This result supports our consideration for the catalyst deactivation observed in the CV and the CA experiments in acidic media. In addition, dissolution of Pt can be another reason for reduced activity of Pt/C catalyst in acidic media. It is reported that degradation of Pt catalyst for the fuel cell electrodes is caused by dissolution redeposition of Pt and acidic condition enhances Pt dissolution [34,35]. On the other hand, electrooxidation of **GC** in alkaline media afforded **OX** and CO_2_ (47.8 and 52.1% Faradaic yield, respectively). To elucidate effects of the applied potential, temperature and alkali cations on the product selectivity, we conducted CA experiment under varied conditions. The CA experiment performed at the applied potential of 1.2 V in 0.5 M **GC** with 20 wt% KOH at 50 °C resulted in 51.7 and 47.5% of Faradaic yields for the formation of **OX** and CO_2_, respectively (Figure 6(c)), although we obtained approximately 100% selectivity for **OX** production under the same conditions in our previous paper [19]. A possible reason for the difference in the product selectivity between the present and the previous experiments is certain differences in Pt/C catalysts used. We are now examining catalytic ability of various metal nanoparticles and nanoalloys for electrochemical **GC** oxidation in ongoing study, and it is becoming clear that subtle difference in catalyst properties, including size, morphology, and composition, greatly affects product selectivities. Figure 6(b)–(d) reveal that the reaction temperature gives greater influence on the product selectivity rather than applied potential. Sitta et al. investigated the effect of alkali cations, such as Li^+^, Na^+^ and K^+^ cations, on the electrocatalytic oxidation of ethylene glycol on a Pt electrode in alkaline media [36]. In this study, we demonstrated that alkali cations made a significant impact on the product selectivity. In the CA experiment using LiOH as an electrolyte, Faradaic yield for **OX** production attained 92.2% (Figure 6(e)), which is approximately twice as high as the yield with K^+^ ions (Figure 6(b)). Garcia-Arez et al. suggested that alkali cations can form an ion pair with anions adsorbed on Pt surface [37]. It is well known that alkali cations forms a cluster-like structure with water molecules (M^+^(H_2_O)_X_) in aqueous solutions, and a larger effective charge of Li^+^ facilitates the formation of a solvated cluster. The non-covalent interaction between OH species adsorbed on the surface, i.e. OH_ad_ and solvated cations inactivates OH_ad_ and blocks the catalyst surface, resulting in reduction of the amount of surface sites needed for the C–C bond breaking [30]. Therefore, the smaller amount of the active surface site in LiOH solution realizes the high selectivity for **OX** production [36]. These results suggest that environment-friendly power generation, i.e. low CO_2_ emission, will be achievable by optimizing catalysts and reaction conditions.

**Figure 7.**
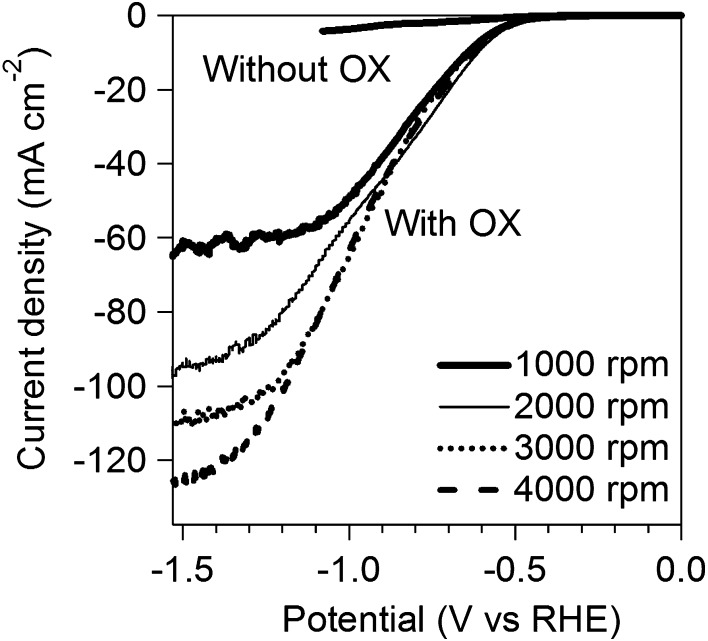
RDE linear sweep voltammograms of TiO_2_ in aqueous solution of 0.2 M Na_2_SO_4_ in the presence and absence of OX with various rotating rates at 50 °C.

**Figure 8.**
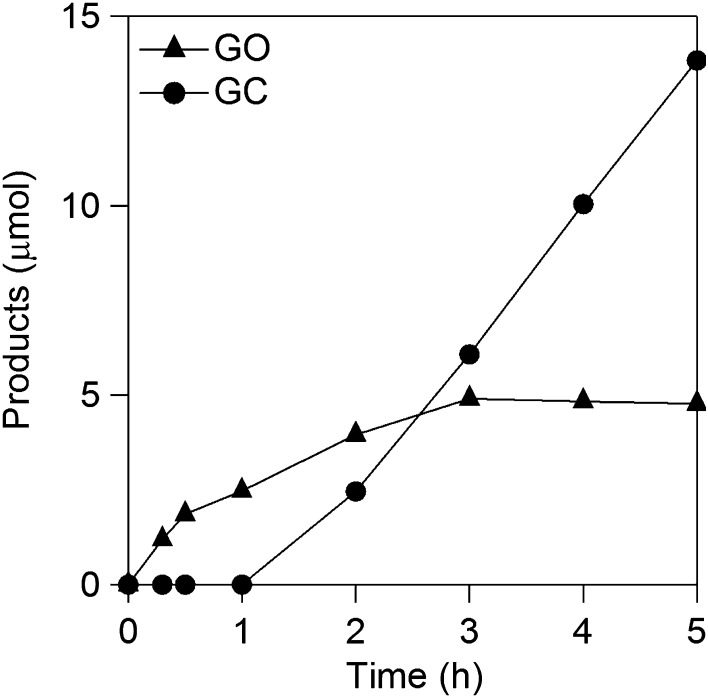
Koutecky–Levich plots at different potentials.

**Figure 9.**
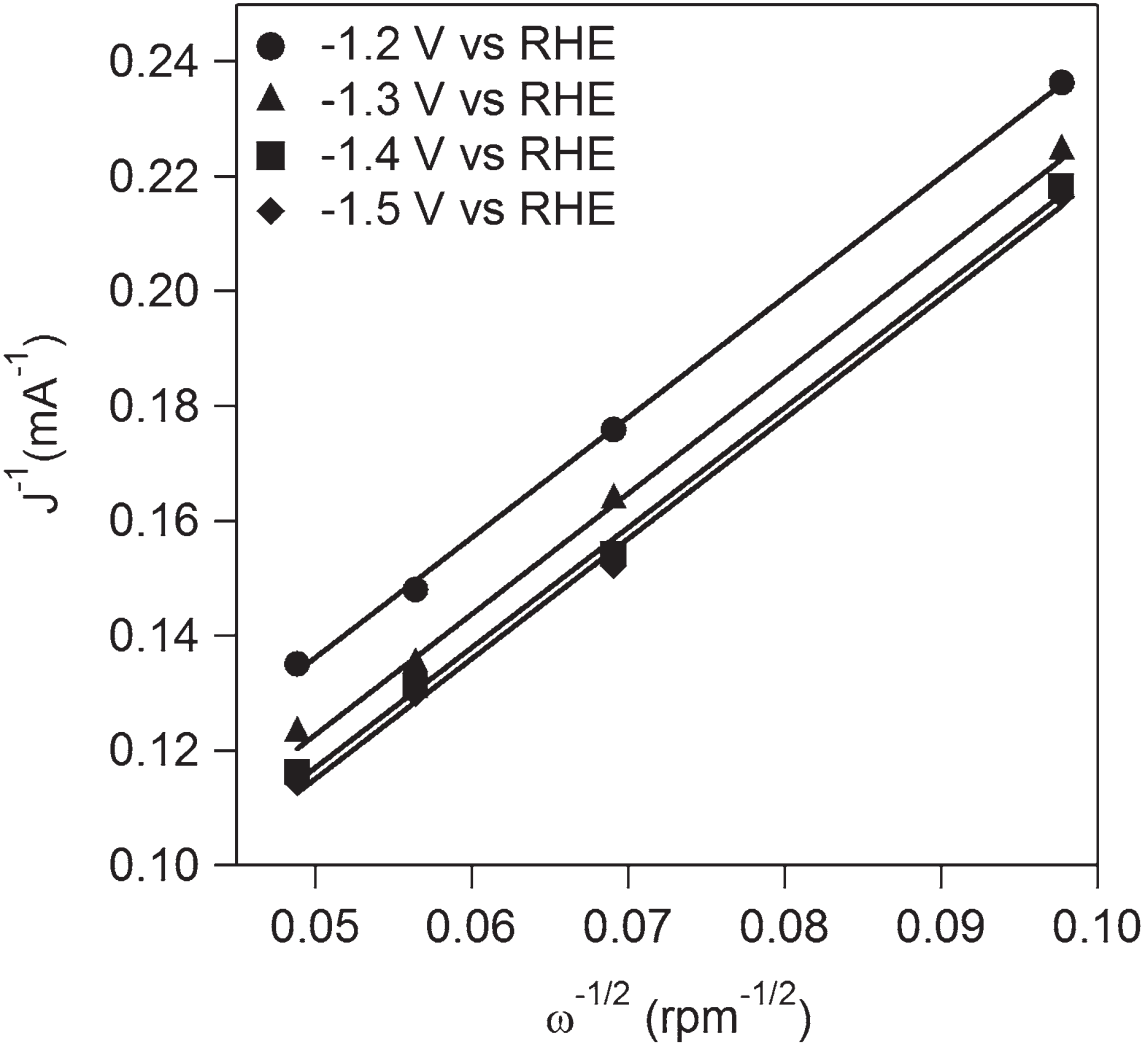
Time courses of GO and **GC** formations in a CA experiment at −1.5 V vs. RHE using RDE.

**Figure 10a.**
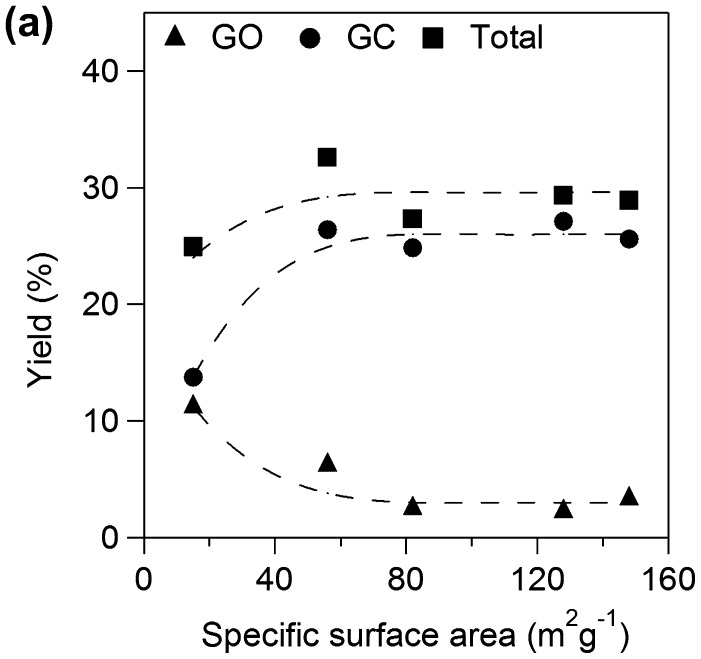
Yields for GO and **GC** against specific surface area of TiO_2_.

**Figure 10b.**
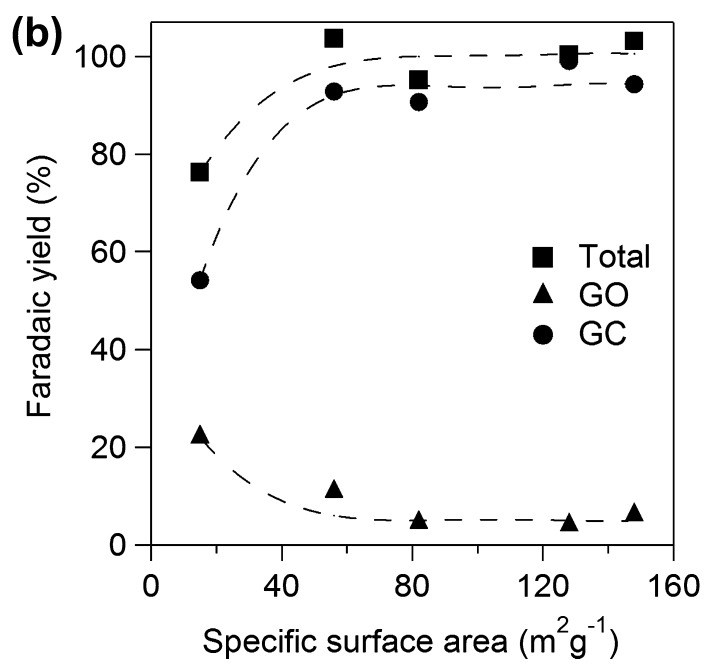
Faradaic yields for GO and **GC** against specific surface area of TiO_2_.

**Figure 11.**
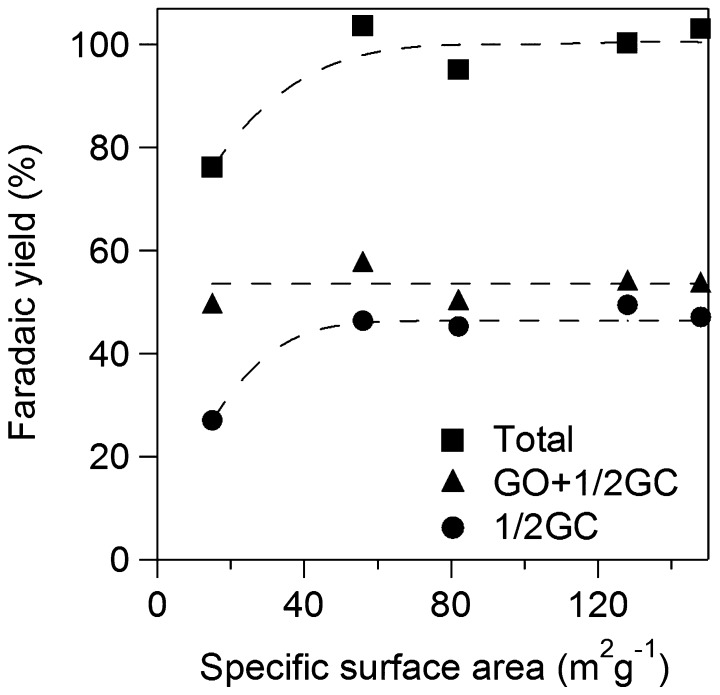
Faradaic yields for GO+1/2**GC** and 1/2**GC** against specific surface area of TiO_2_.

### 3.2. OX reduction

#### 3.2.1. Analysis of kinetics of **OX** reduction

Kinetics of **OX** reduction has not been investigated in detail although some studies for **OX** reduction were reported [19,22]. We performed electrochemical reduction of **OX** using RDE to investigate the kinetics of **OX** reduction. Figure 7 shows linear sweep voltammograms in aqueous solution of Na_2_SO_4_ in the presence and absence of **OX** using RDE with various rotating rate at 50 °C. Steep increase in current density was observed in the presence of **OX** while small current density was observed in the absence of **OX** below –0.3 V vs. RHE. The results indicated that the reductive currents were originated from **OX** reduction below −0.3 V vs. RHE.

The kinetics of **OX** reduction including the electron transfer number (*n*) was analyzed on the basis of the Koutecky–Levich equation. Figure 8 shows Koutecky–Levich plots obtained from the polarization curves at various potentials in Figure 7. The linearity of the Koutecky–Levich plots and near parallelism of the fitting lines suggest the first-order reaction kinetics toward the concentration of **OX** and similar electron transfer numbers for **OX** reduction at different potentials [38]. The *n* values were calculated to be 2.3 at 1.2–1.5 V vs. RHE from the slopes of Koutecky–Levich plots. The n value, 2.3, corresponds to reduction of **OX** to GO. However, the large amount of **GC**, four-electron reduction compound, was observed in the CA experiments using Ti plate electrodes in our previous work [19]. To elucidate the discrepancy, we performed a CA experiment using RDE. Figure 9 shows time courses of the amount of GO and **GC** in a CA experiment using RDE at –1.5 V vs. RHE. Although GO formation was observed in the initial period of the experiment, the amount of **GC** was increased after 1.5 h. Considering that **GC** is detectable after 30 min in CA experiment using a Ti plate electrode in the previous work, the difference in products is probably assignable to the difference in the electrode area, i.e. areas of Ti plate and RDE are 4.0 and 0.071 cm^2^, respectively. Small electrode surface area of RDE causes a long reaction time for reduction of GO to **GC** because GO molecules formed through two-electron reduction of **OX** immediately desorb from TiO_2_ catalysts on RDE and diffuse into solution. On the other hand, reduction of GO to **GC** on a Ti plate relatively easily occurs due to the larger number of catalytic sites located on a Ti plate electrode. From a LSV curve recorded using RDE in 3 min, where only the GO formation proceeds (Figure 7), we obtained 2.3 of the n value, whereas **GC** formation was observed in the experiments using a Ti plate. Time courses of product concentrations, as shown in Figure 9, seem to correspond with product distributions at the initial period on the Ti plate electrode because the area of Ti plate is 56 times larger than that of the RDE and reactions proceed faster on a larger electrode. Based on these results, we concluded that the four-electron reduction of **OX** to **GC** proceeds through successive two-electron reductions as described in Equations (2) and (3). The **OX** is first reduced to GO through two-electron reduction, and then **GC** is produced from GO through further two-electron reduction.

(2) $${\left({\text{CO}}_{\text{2}}\text{H}\right)}_{\text{2}}+2{\text{H}}^{\;\text{+}\;}{\;\text{+ 2 e}}^{-}\to \text{HOOC}-{\text{COH + H}}_{\text{2}}\text{O}$$

(3) $$\text{HOOC}-{\text{COH + 2 H}}^{\;\text{+}\;}{\;\text{+ 2 e}}^{-}\to {\text{HOCH}}_{\text{2}}-{\text{CO}}_{\text{2}}\text{H}$$

#### 3.2.2. Effects of specific surface area of TiO_2_ catalyst on performance of **OX** reduction

We examined effects of specific surface area of TiO_2_ catalyst applied onto Ti plate electrodes for **OX** reduction. Figure 10a shows yields for GO and **GC** using various TiO_2_ electrodes for 2 h. Total yields for GO and **GC** increased with increase in specific surface area up to 60 m^2^ g^−1^ and became almost constant above 60 m^2^ g^−1^. The results suggest that the larger number of active sites contributes to higher yield in the range of small specific surface area and concentration of **OX** can be a rate determining factor on the catalysts having larger surface area above 60 m^2^ g^−1^, i.e. the TiO_2_ cathode having larger specific area would show higher activity for higher concentration of **OX**. Yields of **GC** increased with increase in specific surface area while yield of GO decreased up to 60 m^2^ g^−1^ and both yields also became constant above 60 m^2^ g^−1^. The results suggest that larger specific surface area also contributes to higher selectivity for **GC**. Next, we investigated effects of specific surface area on the product selectivity from the aspect of the amount of electrons consumed for production of GO, **GC,** and H_2_. Electrochemical reduction of **OX** to **GC** proceeded through successive two-electron reductions as described above, and H_2_ production by proton reduction is only regarded as a side reaction [21]. Thus, target reactions in the system were reduction of **OX** to GO, reduction of GO to **GC,** and reduction of protons to H_2_. Figure 10b shows Faradaic yields for GO and **GC** against specific surface area of TiO_2_. Total Faradaic yield of GO for **GC** increased with increase in the specific surface area up to 60 m^2^ g^−1^ and reached almost 100% above 60 m^2^ g^−1^. Faradaic yield of GO decreased and Faradaic yield of **GC** increased with increase in specific surface area. We reconstructed Faradaic yields for GO and **GC** in Figure 10b to investigate product selectivity based on the amount of electrons used for reduction as follows. **GC** is produced from **OX** through GO formation by successive two-electron reductions. Therefore, a half of Faradaic yield for **GC** should be considered to be equal to the Faradaic yield for GO. Figure 11 shows the half of Faradaic yield for **GC** (1/2**GC**) and summation of Faradaic yield for GO and the half of Faradaic yield for **GC** (GO+1/2**GC**) against specific surface area. Faradaic yield for 1/2**GC**, i.e. selectivity for reduction of GO to **GC**, was increased with increase in specific surface area up to 60 m^2^ g^−1^ and became almost constant above 60 m^2^ g^−1^ and Faradaic yield for GO+1/2**GC** i.e. reduction of **OX** to GO was constant around 55% in the all range of specific surface area. H_2_ production occurred below 60 m^2^ g^−1^ specific surface area since total Faradaic yield of GO and **GC** did not reach 100%. The results indicate that reduction of GO to **GC** competed with reduction of proton to H_2_ in the range of small specific surface area and large specific surface area contributed to suppression of proton reduction. Therefore, promotion of GO reduction and suppression of H_2_ production in the range of large specific surface area resulted in high selectivity of **GC** as shown in Figure 10a. Although we cannot determine a definitive factor for the selectivity at this stage, surface area is considered to be one of controlling factors in this system. Based on the results, the TiO_2_ cathode having large specific surface area is appropriate for high yield and selectivity for **GC** in the **OX** reduction system.

## 4. Conclusions

In this work, fundamental experiments on both **GC** oxidation and **OX** reduction were conducted. The CV measurement with various transition metal electrode in aqueous solution containing **GC** demonstrated that Rh, Pd, Ir, and Pt have preferable features as a catalyst for electrochemical oxidation of **GC**, and Pt has the highest catalytic activity. We found that the selective oxidation of **GC** to **OX** on Pt/C more favorably proceeds in alkaline media. In particular, electrooxidation of **GC** in LiOH solution resulted in dominant formation of **OX**, i.e. 92.2% of Faradaic yield. The kinetic study of **OX** reduction clearly indicated that four-electron reduction of **OX** to form **GC** proceeds through two successive two-electron reductions. In addition, it was elucidated that application of TiO_2_ catalysts with large specific area to electrochemical reduction of **OX** can effectively suppress H_2_ production and achieve high selectivity for **OX** reduction. We hope that these findings will contribute not only to improve the energy efficiency for CN circulation but also to enhance catalytic conversion in any other electrochemical reactions using alcohol or acid molecules.

## Disclosure statement

No potential conflict of interest was reported by the authors.

## Funding

This work was supported by Japan Science and Technology Agency – CREST.
